# Polymer Geogrids: A Review of Material, Design and Structure Relationships

**DOI:** 10.3390/ma14164745

**Published:** 2021-08-22

**Authors:** Mohammad Al-Barqawi, Rawan Aqel, Mark Wayne, Hani Titi, Rani Elhajjar

**Affiliations:** 1 College of Engineering & Applied Science, University of Wisconsin-Milwaukee, 3200 N Cramer St, Milwaukee, WI 53211, USA; albarqa2@uwm.edu (M.A.-B.); riaqel@uwm.edu (R.A.); hanititi@uwm.edu (H.T.); 2 Tensar International, Alpharetta, GA 30009, USA; MWayne@tensarcorp.com

**Keywords:** polymer, geosynthetics, geogrid, civil engineering, materials, smart materials

## Abstract

Geogrids are a class of geosynthetic materials made of polymer materials with widespread transportation, infrastructure, and structural applications. Geogrids are now routinely used in soil stabilization applications ranging from reinforcing walls to soil reinforcement below grade or embankments with increased potential for remote-sensing applications. Developments in manufacturing procedures have allowed new geogrid designs to be fabricated in various forms of uniaxial, biaxial, and triaxial configurations. The design flexibility allows deployments based on the load-carrying capacity desired, where biaxial geogrids may be incorporated when loads are applied in both the principal directions. On the other hand, uniaxial geogrids provide higher strength in one direction and are used for mechanically stabilized earth walls. More recently, triaxial geogrids that offer a more quasi-isotropic load capacity in multiple directions have been proposed for base course reinforcement. The variety of structures, polymers, and the geometry of the geogrid materials provide engineers and designers many options for new applications. Still, they also create complexity in terms of selection, characterization, and long-term durability. In this review, advances and current understanding of geogrid materials and their applications to date are presented. A critical analysis of the various geogrid systems, their physical and chemical characteristics are presented with an eye on how these properties impact the short- and long-term properties. The review investigates the approaches to mechanical behavior characterization and how computational methods have been more recently applied to advance our understanding of how these materials perform in the field. Finally, recent applications are presented for remote sensing sub-grade conditions and incorporation of geogrids in composite materials.

## 1. Introduction

Geogrids are a subset of geosynthetic materials typically made from polymeric materials used to reinforce soils, retaining walls, and other sub-surface roads and structures. Geogrids are used to provide solutions when engineers encounter unfavorable soil conditions, allowing a reduced thickness in a pavement structure by stiffening the sub-base. Geogrids are also applied to handle failure scenarios in infrastructure retrofits via a reduced sub-base resulting in thinner asphaltic top layers. According to a report by Allied Market Research, the global geogrid market generated $0.8 billion in 2018 and is expected to reach $1.8 billion by 2026 [1]. This increase is driven by the rise in infrastructure development activities worldwide and the surge in adoption in the construction sector due to ease in handling, environmental safety, and high mechanical properties. The report noted that increased awareness of geogrid and increased research and development activities create new opportunities. The primary sector to see growth will be the road industry representing more than one-third of the total share. Soil reinforcement is also seen as a growth area for using geogrids in road and railways, slopes and earth embankments, foundations, and retaining walls [1]. Geogrid material can vary between knitted or woven grids, non-woven fabrics, and composite fabrics. Geogrids are commonly made from polyester, high-density polypropylenes (PP), or high-density polyethylene although other materials are typically used (Table 1). They are mainly fabricated using extrusion, knitting, weaving, extrusion, and welding. In extruding, punched flat plastic sheets are extruded into the desired final configuration. The final shape configuration is defined by the punching pattern and extrusion parameters, in which each punch will end up as an aperture. In knitting or weaving, resistance in geogrids occurs through a tension mechanism as the reinforced layer is pulled in tension after interlocking with soil or aggregates. Geogrids are typically made from polymers with typically large apertures compared to geotextiles, another commonly known polymer material used in civil engineering applications. Geogrids can be differentiated from geotextiles. Geogrids are mainly used as reinforcing materials, compared to geotextiles with many other non-reinforcing functions such as drainage and filtration. In addition, the geogrid reinforcing mechanism is different due to the interlocking of the soil and aggregate with the grid membrane. Single yarns of polyester or polypropylene are weaved into flexible joints forming the aperture and subsequently coated with bituminous or latex coatings. Single ribs are extruded from polyester or polypropylene material in the extrusion and welding process and later welded together into the desired shape and size [2].

The following manuscript was developed after not finding a comprehensive article in the literature examining the topic of polymer geogrid materials jointly from the material, structure, and characterization parts. Previous review topics on the subject of geogrids had considered more narrowly focused reviews that impact on specific properties, e.g., bearing capacity [3], soil reinforcement [4,5]. There were also reviews focused on specific applications, e.g., retaining walls [6], pavements [7,8] or railroads [9,10]. As the purpose of this review paper is to assess and synthesize the current literature on geogrids to enable new frameworks to emerge such as remote sensing, an integrative review was used as the methodology of this literature review paper. Google Scholar, Science Direct, and EI Compendex (Engineering Village) were the primary search engines investigated. More than 160 papers were collected during this effort and some were not used if they did not fit under the specific sections outlined.

This review presents a literature review on geogrid material technology in the past three decades to further the current state of the science of geogrids development, including uses, main types, performance, efficiency, construction techniques, and further advancements for structural sensing. This review represents a comprehensive look at the literature on geogrids with three sections. The first section on physical and chemical characteristics covers microstructure and environmental behaviors in the primary geogrid products currently on the market (uniaxial, biaxial and triaxial geogrids). Microstructural and environmental behaviors for both physical and mechanical properties, installation damage, and the effects of defects are reviewed. The second section of this review is concerned with the geogrids in the structure and how they behave. Here we look at the effect of soil–geogrid interaction and how geogrids have performed as a reinforcement not only in soil, but also in asphalt, concrete, and retaining wall applications. Beyond the laboratory studies, we assess the approaches and results for in situ assessment of reinforcement and stabilization of soils and structures. Finally, the last section looks at advanced characterization in geogrids and how they have been used to sense subsurface conditions and have been integrated with electrical and optical methods.

## 2. Physical and Geometric Characteristics

The properties of a geogrid depend on the geometric configuration and characteristics of the materials used to manufacture the geogrid. The mechanical properties are significantly influenced by the grid geometry, which includes the aperture size, percent open area, and thickness. The aperture size should be large enough so that the aggregate and soil can penetrate and interlock with the geogrid. The interlock between the geogrid and surrounding soil provides the composite behavior required for soil stabilization. The percent open area of a geogrid is typically 50% [11]. The grid thickness applies for both the rib and the junction thicknesses, which should be thick enough and of adequate rigidity to allow the strike-through of surrounding soil, stone, or other geotechnical material [11]. The geogrid junctions are typically thicker than the ribs. The physical characteristics of the geogrid, such as creep, tensile modulus, junction strength, and flexural rigidity (ASTM D7748) are also of interest to meet the design and serviceability requirements [12,13]. Higher geogrid tensile modulus becomes more critical when loading conditions are more instantaneous [14]. In addition to rib strength, junction strength is also a parameter that is usually considered an indicator of manufacturing quality and can provide information about grid stability and tension reinforcement capability [15]. The flexural rigidity is the resistance of geogrid when undergoing bending and is a good indicator of the propensity of the geogrid to folding or wrinkling [11].

**Figure 1:**
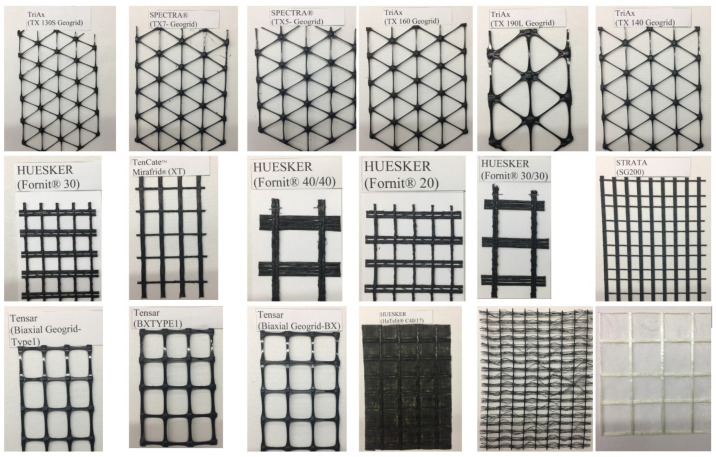
Variety of geogrid materials being produced.

The aperture shape of geogrids heavily influences their mechanical behavior and characteristics. Geogrid samples made from high-strength polyester yarn coated with polyvinyl chloride (PVC) and cured at 180 °C with five different aperture sizes were tested to determine the effect of aperture size and soil on the pullout resistance of the geogrid material [16]. The smaller aperture size resulted in improper interlocking between the soil and geogrid, causing highly scattered results. At larger aperture size, the frictional force between the soil and geogrid decreases. Both extreme cases resulted in a lower pullout resistance. However, the largest pullout resistance was achieved in the soil with the largest particle size due to the increased interlock. In addition, the pullout resistance was more influenced by the density of the ribs in the transverse direction [16]. Results show a direct correlation between aperture size and pullout test results [17]. The maximum interaction between the geogrid and soil is achieved when the aperture size is similar to soil grain size [18]. The properties of four different biaxial geogrid materials used for stabilizing pavement subgrade were studied using accelerated pavement testing (APT) for pullout and shear. APT provides the ability to conduct tests in a short time and control the loading and environmental conditions. The study found that the essential geogrid attributes, when selecting a geogrid, were the dimensions of the aperture, the strength of the geogrid, node strength, and the resistance to bending [17]. Geogrids’ mechanical properties with rectangular and triangular apertures have been reported in the literature [16,19,20,21,22,23,24]. Figure 1 depicts typical geogrids with rectangular and triangular apertures. It was found that in rectangular aperture geogrids, the tensile strength and stiffness are direction-dependent. The uniaxial loading relative to the orientation of the ribs dictated the strength observed. Higher tensile properties were achieved when loading the geogrid in either the machine or cross-machine direction. The tensile strength decreases with orienting the load away from these directions. On the other hand, the triangular aperture size geogrids carried the load uniformity at all loading directions (Table 2). Moreover, the triangular aperture geogrids showed a better distribution of stresses compared to the rectangular geogrids. Consequently, the triangular aperture geogrid appears to be more efficient at carrying off-axis stresses that do not line up with the primary directions. The apparent stiffness is increased due to the ribs present in different planes [20].

Triangular (or triaxial) geogrids are increasingly popular due to the more quasi-isotropic behaviors compared to uniaxial and biaxial grids. The triangular geogrid reinforced base course over a weak subgrade was tested against an unreinforced soil sample under cyclic loading [23]. It was found that the soil reinforced with triangular geogrid achieved a higher traffic benefit ratio. An increase in the traffic benefit ratio was observed at the heavy-duty geogrid. Moreover, the maximum vertical stress and the permanent deformations in the soil decreased with the triangular geogrid reinforcement. The stresses were more uniformly distributed among the soil and geogrid [23].

### 2.1. Junctions and Connections

The junction point in a geogrid is a critical area since the stress concentrations may be amplified. It is a location where there is a thickness transition compared to the ribs and where failures may initiate. Also, in many manufacturing processes, the extrusion process which strengthens the ribs by ordering the polymer chains may not be at play at the junction locations. An experimental study conducted to study the tensile response of biaxial geogrids with integral and welded junctions under biaxial loading showed an increase in geogrid stiffness when loaded biaxially compared to uniaxial loading. Such an observation could be related to the junction response under the principal and orthogonal tensile stresses and strains due to Poisson’s ratio and re-orientation of the amorphous molecules at the nodes [25,26]. A similar study was conducted to evaluate geogrids’ design and specifications parameters, and recommendations were presented [27]. In this study, testing techniques were introduced to characterize the tensile behavior of geogrids ribs and junctions subjected to uniaxial and biaxial tensile loads. Biaxial constant rate of strain (CRS) tests showed higher stiffness than uniaxial CRS tests. Uniaxial sustained loading tests showed higher stiffness than biaxial sustained loading tests; hence, tension testing may not wholly capture the material behavior [27]. Tensile experiments have also been conducted, showing that failure in geogrids tension occurred mainly by the rupture of joints and edges rather than the rib due to material variability in quality [28]. These connections are considered a limiting strength factor. The connection of a geogrid can be achieved either by overlapping/frictional mechanisms or by mechanically connecting the geogrids [29]. Frictional connections depend mainly on the shear strength of the geogrid. In contrast, mechanical connections involve adding additional elements to improve structural rigidity and are usually used in soil-retaining walls [30]. The overlapping or frictional connection is designed to assure proper and adequate overlap lengths. The overlapping length is governed by the geogrid-soil interaction behavior, which pullout tests can experimentally determine. The overlapping length depends mainly on the soil type/California bearing ratio (CBR) value for base reinforcement. Bodkin joints are stiletto-shaped dowel bars for geogrid connections, and are considered the most efficient mechanical connections in providing complete load transfer between both sides of the geogrids [29].

### 2.2. Effect of Loading Direction

**Figure 2:**
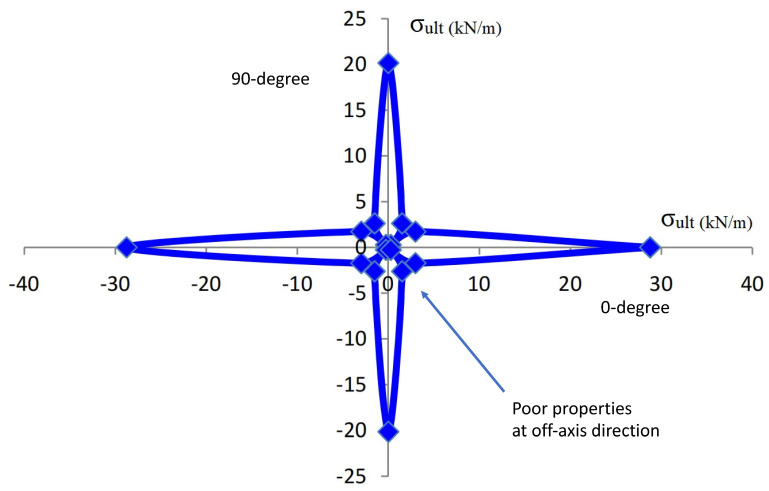
Ultimate tensile strength (kN/m) of tested geogrid specimens around 360 degrees loading directions, ref. [31].

Biaxial geogrids are typically characterized by machine and cross-machine directional properties since the ribs are orientated perpendicular to each other. In practical applications, such as parking lots and traffics on construction sites, the externally applied stresses are multidirectional. The principal stresses do not line up with the orientation of the biaxial ribs as designed. Consequently, it is crucial to look at the biaxial geogrid response when subjected to tensile loads applied in ribs orientation in both orthogonal directions as well as tensile loads applied in directions not following the ribs orientation [31]. A comparison was made by the authors to investigate the geogrid products of a given high volume manufacturer of geogrids that produces different products to the different market segments. Table 2 shows the range of properties and characteristics of uniaxial, biaxial and triaxial geogrids from this one vendor. The values reported concur with a numerical study conducted to study the behavior of biaxial geogrids subjected to tensile loads oriented in different directions [31]. The geogrid was observed to exhibit both the least tensile strength and stiffness when loaded at a 45 degree angle to the machine direction, as shown in Figure 2 [31]. Triaxial geogrids are discussed later in this chapter and show a more quasi-isotropic response.

Numerical investigations conducted using finite element analysis on three-dimensional biaxial geogrids in a piled embankment using truss element, orthotropic, and isotropic approaches show the same strength and stiffness properties in all directions using an isotropic model [32]. In contrast, the orthogonal membrane model exhibits the same strength and stiffness only in two orthogonal directions. In the truss element model, the biaxial geogrid is modeled using truss elements. No significant variation was noticed between the three modeling approaches on SCR (stress concentration ratio) and subsoil settlement. The orthotropic approach results were similar to the truss element approach, while the isotropic approach results were higher for maximum tensile strength. Moreover, increasing the pile spacing, embankment height, and compression index of the soil, improved the apparent geogrid tensile strength [32]. The pile spacing was observed to exhibit the most significant effect. It results in maximum geogrid tension occurring in the pile edge rather than in the middle of pile spacing.

### 2.3. Oxidation, Temperature, and Pressure Effects

Geogrids are exposed to various swings in temperature when at storage facilities, at the site pending installation, or in place. These temperature swings may include sun exposure and freeze–thaw cycles. Moreover, geogrids are typically subjected to different levels of normal stress due to load variations, transportation, expansion, or contraction because of temperature fluctuations. As a result, the behavior of the geogrids subjected to temperature and pressure has been an issue of significant interest [33,34,35,36]. The oxidization resistance in geogrids was studied at various temperatures and pressures using multiple aging times by measuring the geogrids’ melting index and tensile properties. The melt index is a qualitative approach to evaluate the polymer molecular weight and can be used to monitor variations in the molecular weight due to oxidation. Two different uniaxial geogrids were used in the experiment, where one geogrid had a higher unit weight and smaller aperture size in both machine and cross directions. In the first tests, temperature was increased while keeping the pressure unchanged (at atmospheric pressure). In this series, both geogrid types were used, and it was noted that the temperature increase at different aging did not affect the tensile behavior nor the melting index of both types of geogrids. The oxidization induction time was also reported to be reduced to 15% after 84 months of exposure at 75 °C. However, oxidization degradation within a reasonable time was not achievable by the increase of temperature only [36]. Note that long-term degradation of high-density polyethylene (HDPE) and PP is due to the oxidation of polyolefins where is it is hydrolysis for polyesters in polyethylene terephthalate (PET). Additives and developments in polymer chemistry have made significant advancements in reducing the degradation. However, currently the long-term design strength of geogrid strength is typically used to address the long-term degradation. AASHTO guidelines [37] are commonly used which have reduction factors to the ultimate strength of the geogrid for creep, site damage and durability. The factors are typically applied to the tension strength D6637 Method B of the geogrid. The tests used to determine the oxidation reduction are shown in Table 3.

In another series of experiments, only the geogrid with higher unit weight and smaller aperture size was studied under four temperatures and 16 pressure points. Substantial changes in the geogrid’s tensile behavior and melting point were noted after 23 months under 65 °C and 6.3 MPa oxygen pressure. It was also found that the oxidization degradation could be achieved between 2–5 years by the combined effect of oxygen pressure and temperature. The lifetimes were modeled using the Arrhenius equation. The predicted lifetimes at 20 °C site temperature for the two types of geogrid tested under the first series of experiments were found to exceed 120 years for both geogrid types; consequently, the mechanical properties of the geogrids will remain unchanged throughout the services lifetime, which was assumed as a 100-year design life. On the other hand, the predicted lifetime for the geogrid with higher unit weight and smaller aperture size under the combined effects of elevated temperature and high pressures was more than 100 years at 1 atm pressure and 20 °C. The predicted lifetime for both series exceeds 100 years, which is considered the design lifetime for most projects. Moreover, it was also found that oxidization degradation can be significantly accelerated by high oxygen partial pressure, as the latter will accelerate the antioxidant depletion rate [36].

Oxidation effects on PP geogrids were studied using three accelerated oxidation approaches (a) wet autoclave, (b) dry autoclave using pressurized oxygen, and (c) oven aging in normal air. The PP geogrids at ambient conditions had lifetimes of approximately 65 years using the autoclave tests under high oxygen pressure and greater than 2000 years using oven aging. Autoclave and oven aging were not considered equivalent tests for the geogrid material because the life prediction of the autoclave was 40 times less than that of the oven. The shorter life predicted from autoclave testing is due to the non-linear degradation rates depending on oxygen pressure in the autoclave and limited reduction at lower temperatures [38]. It was also observed that the lifetime of geogrids is inversely proportional to temperature, in which a decrease in temperature will exponentially increase the lifetime of a geogrid [38]. Fourier transform infrared (FTIR) spectrometry, digital scanning calorimetry (DSC), and scanning electron microscopy (SEM) were used to investigate the effects of environmental conditions on HDPE geogrid properties aged for 20 years. FTIR and SEM observations showed slight variations between the aged and unaged samples, which can be related to the strong chemical resistance of HDPE geogrids [39]. DSC showed slow crystallization occurring within an aged HDPE geogrid. In general, no significant changes in the mechanical properties were observed between the aged and unaged samples; hence, HDPE geogrids can be effectively used as landfills reinforcement. However, further research is required to study their properties under accelerated aging factors [39].

Some polymers show much more sensitivity to temperature. The tensile behavior in PVC-coated PET biaxial geogrids at various temperatures ranging from 0–80 °C were conducted according to single rib tests [40]. Tensile test results showed a linear decrease rate (about −0.33% per °C) in the UTS of the geogrid when temperature increases; however, the decrease rate slightly increased when going from 60–80 °C [34]. Moreover, the elongation at break was about 11% from 0–60 °C and approximately 10.25% at 80 °C. The reason for that is the glass transition temperature of the PET material; hence, it affects the mechanical behavior of the tested geogrids when the thermal conditions are the same.

### 2.4. Fatigue, Creep, and Strain Rate Effects

The ASTM Standard Terminology for Geosynthetics, D4439, provides the definition of creep which is defined as, “the time-dependent increase in accumulative strain in a material resulting from an applied constant force”. The process in geogrids is triggered by progressively developing fissures that initiate ultimate brittle failure by reducing the intact load-bearing cross-section. The difference between maximum and minimum stresses drastically affects the fatigue strength of the material [41]. Creep is a permanent deformation over time due to constant stress and elevated temperatures characterized by sample elongation. In creep testing, the load is applied to a specific load level maintained constant until the end of the test while deformations are recorded [42]. Typically, ASTM D 5262 is used to evaluate the creep properties of geogrids. This method requires a minimum time of 10,000 h (around 1.14 years) (Table 3); however, this method is questionable when predicting creep behaviors of geogrids for the service life of hundred years. Other methods are used to evaluate creep properties, as presented later in this section [43]. Numerous experimental studies were conducted examining the fatigue [22,23,44,45] and long-term creep effects [34,35,43,46,47,48,49,50,51,52] on geogrid materials. Some of these studies are discussed in this section.

An experimental study and empirical model were developed describing the tensile fatigue behavior of uniaxially tensile-loaded extruded geogrids made from HDPE [44]. The study explored the effects of varying loading parameters, including pre-stressing, dynamic parameters, and the number of cycles on the hysteresis loops. Results showed that increasing the number of cycles improves the hysteretic stiffness; however, it decreased when increasing the loading amplitude. Also, stiffness was reduced during unloading as the number of cycles was increased. Residual strains showed different behaviors due to loading parameter variation. It was concluded that geogrid tensile strength was not affected by cyclic loading history [44].

New non-conventional equipment was developed and presented in [49] built on the platform designed by Franca et al. [48] to study creep effects. Significant adjustments were made to the loading system, including a rotor placed below the equipment to prevent eccentric loading and reinforce the support beam to allow tensile testing. The elongation measurements recorded using a new video camera approach were much lower than the values published in the literature. The video camera method was more suitable for creep testing but not adequate for tensile testing as the recorded elongation values had a low coefficient of variation. The creep response in PVC-coated PET biaxial geogrids under the effect of temperature was studied [34]. Creep tests were conducted according to ASTM D5262 [53] (Table 3) with a load at 65% of the ultimate load. The strain rate and the total creep strain were observed to increase when the temperature increases for the same loading conditions. Moreover, the creep modulus was observed to decrease when the temperature rises. It was also noticed that increasing the creep load and increasing the temperature reduces the specimen’s rupture time. The impact of stress relaxation, which is closely related to creep, on HDPE and polyester geogrids has been experimentally studied [51]. Geogrids were loaded at 40–80% of their ultimate strength for a month till creep rupture occurred. For polyester geogrids, results showed a maximum stress relaxation of 30% of the initial load. In comparison, maximum stress relaxation of 50% of the initial load was observed for the case of HDPE geogrids.

**Figure 3:**
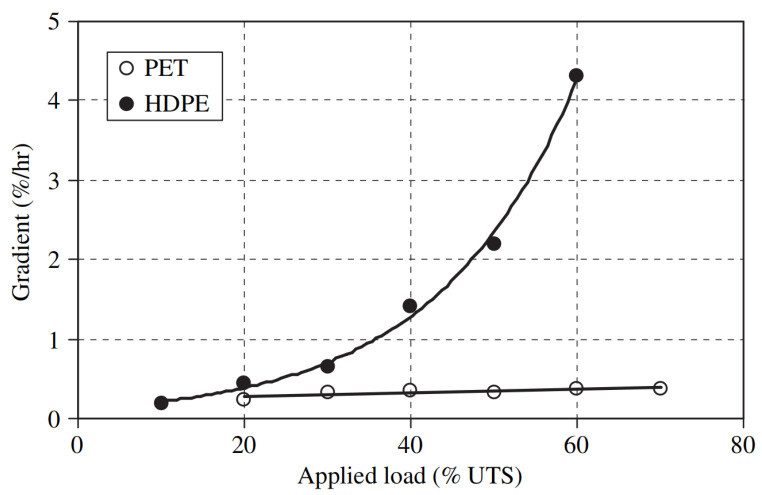
Creep strain rate vs. applied load for high-density polyethylene (HDPE) and polyethylene terephthalate (PET) geogrids [43].

PET provides an advantage over PE and PP in terms of resistance to creep elongation. Time-temperature superposition and isothermal methods were explored on HDPE and PET geogrids [43]. It was observed that the HDPE geogrid had a significantly higher strain than the PET geogrid (Figure 3). In addition, the creep strain rate of HDPE geogrid in the primary phase exponentially increased with increasing the applied load. In contrast, the same rate in the PET geogrid was observed to be independent of the applied load. All three creep stages were seen in HDPE geogrids, while only primary and tertiary stages were observed in the PET geogrids. The superior creep performance in PET is related to the higher glass transition temperatures. HDPE at normal temperatures are operating in the rubbery polymer state and act like a viscous fluid.

Moreover, numerous studies were conducted to capture the effect of strain rate on the geogrids’ mechanical response. For instance, geogrids’ axial and lateral tensile behavior was studied using a video extensometer device for measuring strains at various strain rates [54]. In this study, three types of geogrid were considered: PET, biaxial PP, and uniaxial HDPE. The lateral strains induced in PP and HDPE geogrids were observed to be significantly larger than PET geogrid. The specimen aspect ratio was noticed to not affect the tensile response axially and laterally for the PET and HDPE geogrids, while the PP geogrid having an aspect ratio of one exhibited larger strain values than other aspect ratios. HDPE and PP geogrids’ tensile strength and stiffness increased with increasing strain rate [54]. The same conclusion on strain rate effect on the geogrid tensile strength and stiffness was observed in another experimental study conducted on HDPE geogrids [55]. In addition, the effect of strain rate was slightly smaller in geogrids with high tensile strength. Another experimental study on strain rate was conducted to examine the residual deformation of geogrids subjected to sustained and cyclic loads. The residual deformation followed a hyperbolic response due to the geogrid viscous behavior [56].

The strain rate effect on the tensile behavior of geogrids was also studied [57], in which tests have been conducted according to ISO 10319 standard [58] considering both single rib and wide-rib specimens at six different strain rates. The study’s objective was to develop a valid method for single rib testing that can be closely matched with the wide-rib test without the need to conduct the wide-width test. Different strain rates have been explored, and it has been observed that the geogrid tensile strength increases with increasing the strain rate for both single rib and wide width specimens. The results obtained from single rib testing using a 100 mm/min strain rate were found to be in good agreement with results obtained from the wide-width testing using a strain rate of 25 mm/min; hence, the single rib testing method was considered valid. However, concerns related to single rib failure at a lower elongation strain than the wide-with method still exist, which hampers the use of the single rib method as many specifications use the failure load at a certain elongation level.

### 2.5. Installation Damage and Effects of Defects

**Figure 4:**
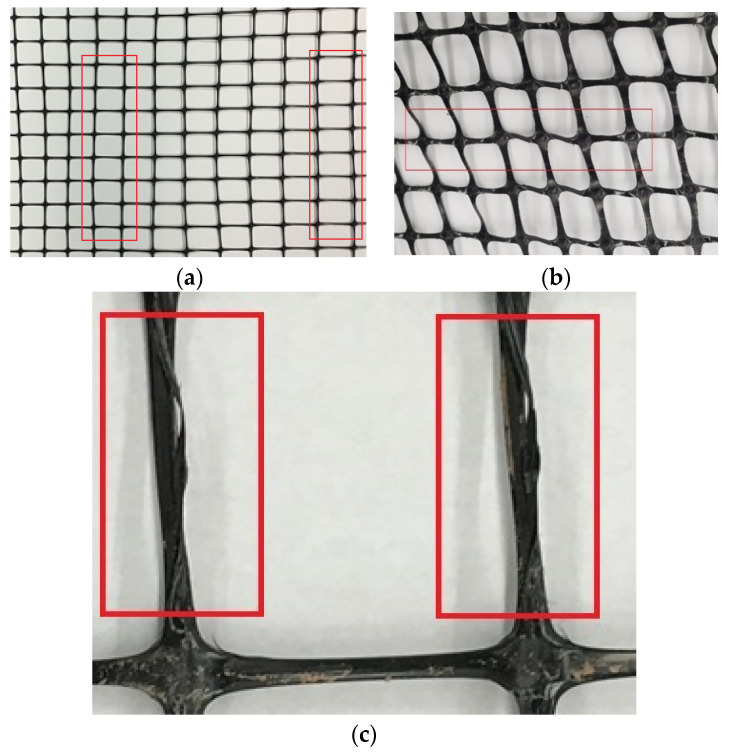
Manufacturing defects in geogrids: (**a**) Variability in aperture sizes, (**b**) bent ribs or variation in aperture shape, (**c**) splitting in geogrid ribs.

Defects and damages are significantly critical as they adversely affect the performance and mechanical properties of geogrids. Causes for such damages could be from manufacturing, shipment, and storage, as well as installation. Manufacturing defects are widespread and essential to be considered in geogrids. Such defects include punctures, tears, flaws, bent ribs, and variability in aperture sizes. Manufacturers conduct thorough inspections on geogrids after manufacturing and before shipment as part of quality control checks because defected geogrids are subject to being rejected by clients such as the departments of transportation (DOTs). Figure 4 shows some of these defects.

Damages could also occur during shipment and storage. As part of quality control, geogrids should be protected from direct sunlight, ultraviolet rays, temperatures greater than 160 °F (71 °C), flames, including welding sparks, mud, dirt, dust, and debris. Also, geogrids should be kept in dry storage and not in direct contact with the ground [59]. As for damage occurring during installation, the leading cause for such damages is abrasion, referred to as the friction (cyclic relative motion) between the contact subgrade and the geogrid [60]. Mainly, abrasion can be classified into two types: (a) abrasion damage from placement and overlaying the fill material [61,62], and (b) time-dependent abrasion damage during the service life of the installed geogrid [18].

The geogrid structure will impact the extent of time-dependent abrasion damage, mechanical damage on the physical, hydraulic and mechanical characteristics [60]. It has been observed from the test results that the impact of abrasion and mechanical damage are highly dependent on the structure of the geosynthetic. Moreover, the strength loss resulting from the abrasion damage is significantly higher than loss due to induced mechanical damage [60]; hence, abrasion damage is the most dominant type of damage affecting the tensile strength of geosynthetics [60]. Geosynthetics permittivity was not affected; however, their aperture size increased, resulting from test setup differences. UV degradation is a major concern as regards the exposure of the geogrid for a certain period of time prior to application under the soil. The guidance for geogrids used in road construction (primary application area for triaxial geogrid) requires 50% retention at 500 h. The values in Table 2 are reflective of properties required rather than the actual material behavior.

The move towards using more construction and demolition (C&D) waste as alternative backfill material has led to some concern on the impact of C&D waste on geogrids. The effect of C&D waste material surrounding the geogrid on the short-term tensile behavior of geogrids has been examined [63]. Intact, exhumed, and mechanically damaged geogrids were tested using microscopy and mechanical methods. The exhumed specimens had tiny cavities and grooves compared to more minor irregularities in the specimens not embedded in the C&D waste specimens leading to higher degradation in strength and stiffness. The strength loss was 2% in the exhumed specimens and 6% in the mechanically damaged samples after being buried for 12 months. [63]. Pullout resistance of geogrids in C&D waste has also been investigated and showed the feasibility of using geogrids with C&D waste after considering confining pressure, specimen size and displacement rates [64]. The pullout interaction coefficients were generally greater than or equal to the values reported in the literature for soil-geogrid and recycled material-geogrid interfaces.

### 2.6. Coatings

Coatings have been proposed to improve the properties of geogrid materials in tension, fatigue, and shear resistance between layers. Coatings can lead to increases in the lifetime of geogrids as well as a reduced maintenance cost. Asphalt emulsion, thermosetting epoxy resin, and acrylic emulsion latex coatings have previously been proposed [65,66,67,68]. These coating can be applied in the manufacturing facility or during the installation of geogrids. Fiberglass geogrid road reinforcements were investigated with these coatings to improve the adhesion properties between asphalt layers and grids [65]. Two kinds of coatings (a polymer-based coating and asphalt emulsion) were applied on single end glass woven, knitted fabrics using glass woven (grids), and a composite fabric (grid + nonwoven) material. Improvement of the fiberglass tensile performance was achieved before and after simulating construction conditions in the field, regardless of the coating used [65]. In another study on fiberglass coatings, it was found that thermosetting epoxy resin coatings resulted in higher tensile pull-off strengths. This difference results from the difference in adhesion of the different coating materials with the geogrid surrounding material, including asphalt binders [66]. In the same study, a reduction in the inter-layer shear resistance was observed with the coatings compared to the unreinforced specimen. However, epoxy resin with sand coating achieved the lowest reduction in the shear strength between layers compared to the unreinforced sample. In the four-point bending tests, cyclic resistance was improved for the double layer systems compared to the unreinforced ones. The same coating used to maximize the shear resistance achieved the highest number of cycles to reach flex point [66].

Another study on coatings of four commercially available geogrids revealed that ethylene/vinyl acetate copolymer (EVAC) and acrylic emulation latex contribute to enhanced physical properties. Such observation could be related to the thin coating thickness and the low glass transmission temperature that is typically less than the geotechnical environment resulting in a much lower coating strength and stiffness than the PET. Moreover, it was found that voids in the geogrid structure will allow water to move through the PET and result in early failure due to coating degradation [67].

The mechanical properties of nonwoven and geogrid composites were evaluated using emulsion impregnation for paving geosynthetic applications. No maximum strain reduction was observed in non-woven geotextiles; however, a significant decrease of the same was observed in emulsion-impregnated geogrid composites. The ultimate tensile strength increased up to 62% in all geosynthetics with a considerable increase in stiffness [68]. Moreover, impregnation caused a decrease in the material’s ability to transmit fluid through pore spaces and fractures, resulting in a lower hydraulic conductivity [68].

## 3. In Situ Behavior and Durability of Geogrid Reinforcement

In the previous section, we reviewed the behavior of geogrids in lab size specimens in environments that are not always representative of the field conditions. Numerous studies have been conducted to study the in situ behavior of geogrids. An experimental study using plate load tests was conducted on sand reinforced with geogrids [69]. When considering the effect of geogrid reinforcement in the soil, an increase in stiffness and plate load-carrying capacity was observed at low displacements. Also, the ratio of the top layer spacing to the plate width was studied, and results showed that decreasing this ratio will enhance the load-carrying capacity of the soil due to the increase in bearing capacity ratio (BCR) [69]. BCR is defined as the ratio of the ultimate bearing capacity of reinforced soil to the ultimate bearing capacity of unreinforced soil.

The effect of a biaxial polypropylene geogrid in tension on the granular base strength was experimentally studied [70]. Four reinforced soil samples with various CBR values have been considered. The conducted penetration tests show that geogrid embedment increases the CBR, especially for those soil samples with low CBR values compared to higher CBR soil samples. Moreover, enhanced stress distribution and decreased soil penetration were noticed, consequently achieving better dynamic loading resistance [70]. Another numerical study was conducted on stresses between the sand and geogrid via a particle flow approach [71,72]. Numerical compound tensile tests, in which the models were calibrated by the direct numerical shear and tensile tests, were able to show load transfer and distributions of displacements and forces in the geogrid at various clamping displacements. Changes in the contact force distributions and orientations were observed because of the load transfer from geogrid to sand by the frictional resistance.

Stress analyses on bases reinforced with a polypropylene triangular-aperture geogrid over weak subgrade under cyclic loading [23] show that the maximum vertical stress at the interface between the base and subgrade increased with increasing cycles. In comparison with unreinforced bases, triangular-aperture geogrids enhanced the overall mechanical performance of reinforced bases. Reduction of maximum normal stresses on the subgrade was observed when using the triangular geogrid reinforcement. Moreover, a decrease in the modulus ratio reduction rate of reinforced bases was noticed compared with unreinforced bases. The bases’ mechanical behavior under cyclic loading was observed to significantly improve when using thicker geogrids with enhanced mechanical properties [23].

A comparative study of the mechanical performance between flexible pavement sections reinforced with multi-axial geogrid and thicker unreinforced flexible pavement sections was examined using large full-size sections [73]. Two full-size specimens with geogrids were dynamically tested with loads simulating highway loading. The pressure values in the subgrade and base earth were equivalent for both the reinforced and the thicker unreinforced pavement sections. The measured subgrade deflections and tensile strains in the unreinforced sections were significantly higher than the reinforced sections; hence, enhancing the stabilized pavement mechanical performance [73].

Field testing of a section of a highway project constructed with an aggregate base course layer reinforced with multi-axial triangular aperture geogrid was also investigated [74]. Modification to the original design included replacing the cement-treated granular base with a geogrid-based design. The International Roughness Index (IRI) is a standardized measure of the pavement roughness which represents ride quality and serves as an indicator for vertical disturbances in the road profile. IRI data were collected using a smartphone-based technology called the TotalPave to evaluate the effect of traffic and climate fluctuations on both reinforced and unreinforced segments. After two years of service, the collected information showed that the average IRI of the geogrid reinforced section was 14% less than the unreinforced pavement. Pavement roughness was also observed to be uniformly distributed along the reinforced pavement section; hence, the addition of a geogrid as a reinforcement material appears to offer optimized pavement designs with extended service life [74]. Another study has been conducted on the same project to verify the target design resilient modulus values for each pavement layer, including the geogrid reinforced aggregate base layer using automated plate load testing (APLT) under cyclic loads [75]. The study represents the APLT testing procedure and results in what appears to be a valid verification testing technique that can be used in various engineering projects.

Poor pre-existing subgrade conditions can be overcome with the proper geogrid application. For instance, a subgrade was constructed using a potentially frozen backfill which raised pavement constructability and long-term performance issues to the Nova Scotia Transportation and Infrastructure Renewal Department [76]. The Department decided to select a design that incorporates multi-axial geogrid as a reinforcement material to optimize the pavement performance. Subgrade and reinforced aggregate layer resilient moduli results generated from the APLT were in conformity with project specifications. As a result, the design was approved for meeting the foundation stiffness requirements without compromising the required highway structural number or height limitations [76].

Triaxial geogrids can be used to overcome expansive soil problems. Expansive soil occurs when the additional water causes significant volume changes, with the clay particles in the soil able to grow 15 times their original size when expanding. This growth in size causes strains in the asphalt pavement above the soil to crack and heave, leading to water seepage that reduces the lifetime of the asphalt pavement. The geogrid materials overcome the effect of expansive soil by mechanically stabilizing the aggregate base via creating a stiffer base for the asphalt pavement. The geogrid acts to separate the subgrade and hence stop its slipping into other base layers. Moreover, it will allow water to move through it and not through the soil and sub-base particles, reducing the soil expansion effect [77,78]. Besides, centrifuge models with geogrids used as reinforcements for soil walls with marginal backfill considering the impact of chimney sand drain [79] show that catastrophic failure can occur in soil walls reinforced with low stiffness geogrids due to the excess pore water pressure. In comparison, the soil wall reinforced with higher stiffness geogrids exhibited better performance.

### 3.1. Effect of Soil–Geogrid Interaction

Static pullout tests were conducted on extruded mono-oriented HDPE geogrids investigating the effect of geogrid length and vertical effective stress on soil–geogrid interaction [80]. The tensile strength obtained from the standard in-air tensile testing procedure was approximately similar to the tensile strength related to the conducted pullout test; thus, it was concluded that the geogrid tensile strength is not affected by the soil confinement. Moreover, strain-hardening behavior was observed in specimens with high confining stress, and consequently higher peak pullout strength due to their extensibility. By contrast, short specimens with lower confining stress exhibited strain-softening behavior.

In another study, a new analytical model was presented to describe the load, and displacement transfer mechanisms along the length of geogrids under pullout conditions [81] using a similar rheological approach demonstrated in [82,83]. When a geogrid is under pullout condition, the longitudinal ribs provide the tensile resistance, while the transverse ribs provide the passive resistance. In their model [81], the geogrids included rheological units consisting of two types of element: friction elements for the shear at the interface between the soil and geogrid and spring elements for the geogrids tensile elongation. The parameters included in the model have been estimated from direct shear tests conducted on soil samples and tensile tests performed on geogrids according to ASTM-4595 [84]. The results generated from the model have been observed to provide a reasonable estimate of the load and displacement transfer along the geogrid as they were compared and found to be in good agreement with the conducted pullout test results.

**Figure 5:**
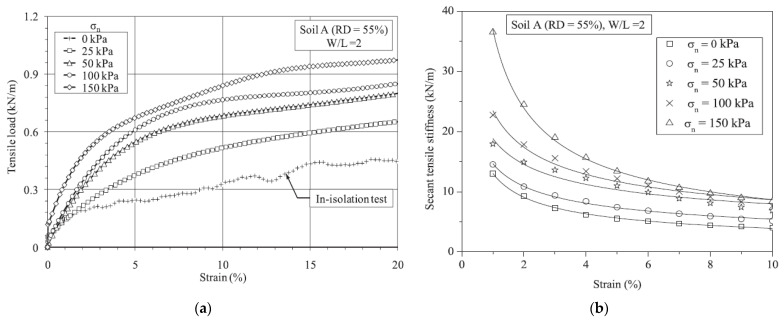
(**a**) Effect of normal stress on the: (**a**) tensile strength of geogrid (**b**) secant tensile stiffness of geogrid [85].

The tensile load-strain behavior of geogrids has been experimentally studied by conducting in-soil tensile testing [85]. Normal stresses, sand-sandwiched layer presence, and soil type effect on the tensile behavior of geogrids were investigated. An increase in the tensile load and the secant tensile stiffness have been observed due to normal stress and geogrid confinement in the soil, as shown in Figure 5 [85]. Moreover, it has been observed that the secant tensile stiffness of geogrids placed in granular soil is higher than those placed in marginal compacted soil with optimal moisture content. The effect of sandwich-layered soil has been observed to enhance the tensile behavior of geogrids placed in marginal compacted soil with optimal moisture content.

Numerical studies conducted to determine the optimal design tensile strength for geogrids to reinforce embankments show that increasing the strength of the embedded geogrid reinforcement increases the safety factor used for the design of geogrids [86]. Due to the small displacements of soil and geogrid, their effect was neglected when determining the optimal tensile strength, which was mainly controlled by the geogrid-soil contact shear stress and the factor of safety. The optimal design was observed to be cost-effective as it enhances the factor of safety used in the design and allows the use of geogrids in soils that do not exhibit superior mechanical properties due to the more appropriate stress distribution across the soil layers. The optimal tensile strength of a geogrid can be obtained by numerical analysis using stress safety factor, stress distribution, displacement, and displacement gap of soil-geogrid, along with the location of geogrid. The displacement and displacement gap between the reinforcing geogrid and the adjacent soil increases as the reinforcement strength decreases below the optimal tensile strength [86]. The Minnesota Department of Transportation has released new software that simulates field tests of stiffness and resilience in pavements [87]. It can also compare the results of the same for pavement over bases with and without geogrids. This software allows designers to include geogrid properties in their calculations when designing a pavement. The new modeling capabilities allow testing various geogrid parameters such as shape and thickness of ribs, aperture size and configuration, moisture content, and aggregate roughness and gradation. The geogrid and aggregate models were simulated via dynamic cone penetrometer (DCP) and lightweight deflectometer (LWD) tests [88]. The test results showed 1.5–2.5 times (depending on moisture content and time of the year) of the resiliency of bases reinforced with geogrid compared to non-geogrid reinforced bases.

### 3.2. Reinforcement in Asphalt, Concrete, and Retaining Wall Applications

The loss of functional and structural properties of pavements nowadays is prevalent due to the high traffic volumes on road infrastructures. Geogrid reinforcement can be introduced in pavements to improve their mechanical performance. In some other applications, geogrids can reinforce thin concrete sections such as Portland Cement Concrete (PCC) pavement overlays [89] to provide post-cracking ductility and higher load-bearing capacity of concrete. This section of the paper reviews the work done on studying the behavior of geogrids as reinforcement material for asphalt and concrete applications. Experiments were conducted to assess the mechanical behavior of fiberglass geogrids covered with thermosetting resin (vinyl-ester) in flexible pavements considering four-point bending with repeated loading cycles and interlayer shear [90]. Results showed that the shear is a crucial factor to consider. The interlayer shear behavior in the geogrid caused a reduction in the interlayer resistance due to the debonding effect between the contact layers [91,92]. The geogrid provided enhanced resistance for the repeated loading cycles as observed in the four-point bending test. It was concluded that fiberglass geogrids embedded in double-layered asphalt concrete pavements enhance the mechanical performance of the system with regards to resistance to repeated loading resulting in longer service life, despite creating debonding at the interface.

In concrete reinforcement applications, the flexural response of concrete beams reinforced with geogrids was studied by conducting four-point bending experimental tests [89]. Tested beams have been observed to exhibit ductile post cracking behavior and higher fracture energy and flexural strength. Concrete compressive strength was observed not to affect the flexural behavior of beams reinforced with uniaxial and biaxial geogrids; however, it influenced beams reinforced with triaxial geogrids as brittle failure was noticed with a drastic increase in deflection [89]. Besides this effort, a similar study was conducted to monitor the behavior of geogrids in concrete beams subjected to static flexural loading by attaching strain gauges to the embedded geogrids [93]. The test results showed that the geogrid was triggered just after applying the load and underwent large deformation after cracking and before failure. Strain measurements of the embedded geogrid showed no slippage or pullout between the concrete and geogrid [93].

The crack resistance and plastic flow resistance of the asphalt concrete is different than in soils [94]. A general creep behavior model was used to analyze the plastic flow behavior. The results showed a significant increase in asphalt concrete viscosity because of geogrid embedment, thus improved adhesion and durability. It was also observed that the durability increases when decreasing the geogrid aperture size due to a reduction in the stress concertation caused by the wheel loading [94]. As for retaining wall applications, field tests on the durability of concrete retaining walls facing geogrid reinforced soil were assessed during construction and post-construction phases [95]. Collected data were analyzed, and a non-linear vertical foundation pressure along the geogrid reinforcement was observed with a maximum value of the vertical pressure occurring at the central part of the geogrid. In addition, the lateral earth pressure within the reinforced soil was observed to be non-linear in result [95].

Concern for long-term effects in geogrids is a major theme in many studies. Durability of polyethylene geogrids for retaining wall applications was examined in a demonstration experiment after eleven years of service in the southwest of the United States [96]. During construction, the selected panels were instrumented with devices to monitor soil and geogrid stresses. These wall panels were exposed to severe heating and pressure. After 11 years of service testing, samples showed the polyethylene geogrids having minimal mechanical or physical degradation [96]. Simultaneously, the tallest reinforced soil slope (RSS) in Southern California was revisited after 11 years to retrieve samples of geogrids used as reinforcement and compare newly produced samples. The reinforcement of these slopes included uniaxial geogrids for primary reinforcement and biaxial geogrids for secondary reinforcement to provide surficial stability. The retrieved samples of uniaxial and biaxial geogrids where nearly identical when compared to the newly produced samples. A high concentration of transition metals was found in the soil, which also had no impact on the geogrids’ properties, despite concerns that a high concentration of transition metals in soils have an accelerating effect on oxidation degradation of polyolefins [97,98]. Another study on the mechanical properties of a reinforced earth wall after 36 years showed minimal degradation in properties [99]. The tensile strength of the extracted specimens was surprisingly higher, perhaps due to the stiffening from field service. The wall performance was remarkable after many years despite being built in a harsh environment.

## 4. Advanced Characterization and Sensing

### 4.1. Non-Contact Imaging Methods

**Figure 6:**
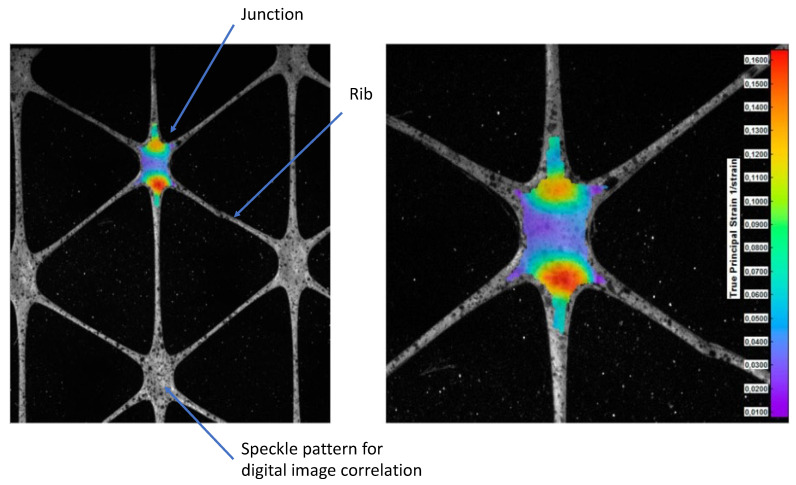
Geogrid components showing stress results from digital image correlation from ref. [101].

A novel technique was developed to measure deformations and local strains for geogrids subjected to tensile loading at various strain rates [100]. Biaxial propylene geogrids, knitted polyester geogrids, and uniaxially extruded HDPE geogrids were studied. Displacements were measured using a video extensometer. Results confirmed the reliability of using such a tool to measure local strains at high resolutions and point out the non-uniform axial and lateral strains distribution of the tested geogrid. Studies on mechanical properties of hexagonal (triaxial) geogrids made of polypropylene [101] using digital image correlation (DIC) were applied in measuring deformations in geogrids subjected to wide-width tensile loading. The ability of DIC to measure deformations at any point in any direction to obtain the principal strains along with their principal directions was presented in their study and proved to be more reliable than standard methods. DIC gives more accurate strain values in comparison with extensometer methods especially when the specimen breaks. The maximum principal strains were observed to occur on node or junction edges, as shown in Figure 6 [101], which justifies the crack propagation type of failure in the specimens [101].

### 4.2. Electrical Resistance Methods

PVC polymer with a filler of carbon black (PVC/CB) has shown the possibility of dual-use in the coating of woven and knitted geogrids to protect against ultraviolet (UV) rays [102] and has also shown promise in providing a conductive medium for sensor-enabled geogrids (SEGG) [103]. These composites possessing electrical conducting particles can create a tensor-resistivity property in the geogrid [103,104,105,106], where the electrical conductivity is seen to change with strain. The sensors can measure the strain in the geogrid up to 20%. Higher sensitivity appears to be related to filler [107] configuration and entanglement of the particles where CB particles because of grape-like formations achieve better properties than carbon nanotubes (CNT) [108]. Molecular dynamic simulations have also been used to study fillers’ impact on sensing abilities, although mechanical verification of such simulations has not been verified experimentally [108]. Experiments where confining pressure was included in the tensor-resistivity measurement seen to show increased variability in the response [103]. The resistance changes in sensor-enabled geogrids were also studied in HDPE and showed resistance to growing very slowly in the beginning, followed by an increase in the slope at the higher temperature with improved sensitivity [33]. Application of graphene on PP and polyester has shown less variability in PP albeit with lower sensitivity to strain. Increased sensitivity was seen with smaller gage lengths and with slower strain rates which is more likely to represent field conditions where geogrids are applied [109].

### 4.3. Fiber Optic Methods

**Figure 7:**
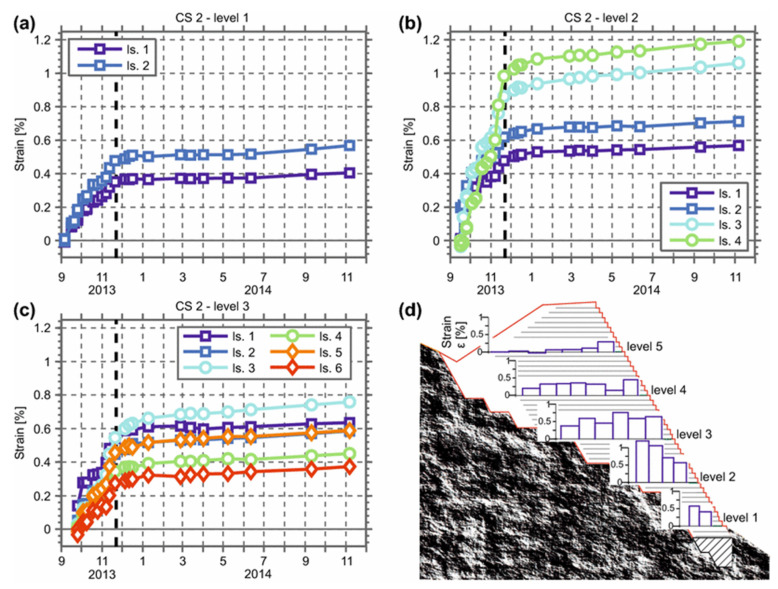
Strain evolution at various levels with the vertical bold dashed line representing the end of construction (**a**–**c**). In (**d**) is the accumulated strain inside the reinforced structure at different levels (Creative Commons CC BY, ref. [116]).

Fiber optic methods have been proposed for long-term measurements of geosynthetic materials [110,111,112,113]. Optical time-domain techniques were applied to monitor the soil slope by bonding the optical cables to geogrids planted under soils [107]. The fiber optic technique showed that strains could be remotely measured while locating the regions where damage may have occurred [114]. Fiber Bragg Gratings (FBG) were also explored for measuring strains in tunnel excavation and long-term monitoring applications, as shown in Figure 7 [115,116]. The FBG sensors captured increasing strains and were shown to survive after one year. Simulations were conducted showing PVC coatings performing better than nylon ones and the location of the fiber optic cable within the center of the geogrid showing more uniform responses. Another fiber-based optical technique uses light backscattering in fibers experiencing strain using techniques like optical time-domain reflectometry. Polymer optical fibers can be used to measure strain up to 40% compared to traditional silica-based fibers that are limited to approximately 1% strain [117]. Optical fibers from poly(methyl methacrylate) (PMMA) have shown remarkable ability to identify the edge of the slope experiencing creep [117]. Vertical walls reinforced using geogrids were also monitored using fiber optic sensors. The sensors measure strain and deformation in the soil walls during and after construction. The results can be used to establish stability ratings based on the peak strains measured [114]. Soil–geogrid interaction was also studied with FBG sensors [118]. Density, initial stress, and boundary conditions were assessed and showed the influence of the boundary on the peak stress propagation from the load applied to the boundary before the pullout. Higher density and initial normal stress result in a slower progression of the shear stress towards the boundary [118]. Numerical modeling and experimental results show that the radial pressure in some sections acting on a buried pipe can also be measured using activated geogrid [119]. The results were also more pronounced with greater normal loads as the soil–geogrid interaction is increased. Distributed sensors for temperature and moisture were used with geogrids to measure the health of a bioreactor system [120]. The measurements can be used to monitor the aerobic decomposition process inside the reactor. FBG sensors were also proposed to measure the displacement in rail ballast for the purpose of preventing track misalignment [121,122,123]. Moreover, FBG sensing technology was also used to analyze geogrid-reinforced sand slope stability as FBG sensors were installed to effectively measure the strain distribution of the geogrid [115]. Limited work has been performed to understand how the hydrolysis resistance [124] may impact the combination of FBG and geogrid combination (Table 3).

## 5. Conclusions

This review highlighted recent work on geogrid behaviors and future opportunities. The geogrids examined are made from polymer materials and are generally classified as either uniaxial, biaxial or triaxial based on the aperture geometry. The research illustrates that geogrids can serve an important role in infrastructure applications and smart materials, simultaneously meeting significant structural requirements and provide actionable information about structural health conditions. The main conclusions of our review can be summarized in the following points:

- Effects of aperture shapes, loading directions, oxidation, temperature, and pressure on geogrids were reviewed and have shown how they significantly influence the characteristics and performance of geogrids. The impact of material selection shows a wide variety of options available and more opportunities exists to improve the creep resistance of the materials having a low glass transition temperature.
- Uniaxial, biaxial and triaxial geogrids have distinct advantages but more guidance is needed from the manufacturers on when to use which material because of the variety of options possible. Customization may be a feasible option to consider.
- A review of the mechanical testing conducted on geogrids (Table 3), including tension, fatigue, creep, and strain rate effect studies, has shown how the geometry impacts the mechanical response of geogrids and the material type. The testing standards need to be better consolidated to reduce the number and type of testing needed to characterize geogrid materials.
- The effect of defects and installation damage on geogrids is a growing area of research illustrating the need to better understand the impact of such variables on the long-term behavior of the geogrids. In particular, when using geogrids with C&D waste is an area needing more research although initial work shows encouraging results. With the increase in geogrid demand, concerns about waste production and using more recycled content will become more pressing. Right now, limited recycled products are being used.
- In terms of long-term and novel applications, geogrids have been successfully used to manage the stresses on buried pipes and similar applications. The addition of fillers to introduce the tensor-resistivity property in the geogrid is an area needing further investigation given the possibility that the additives can reduce the mechanical properties such as tensile strength or ductility.
- The effects of moisture, conductivity, and stress state on the geogrid are also areas not adequately addressed in the literature. Geogrids in sub-surface conditions experience biaxial stress states and exposure to environments where the moisture levels may fluctuate. New testing incorporating multiple variables is needed to reduce the testing scope.
- While fiber optics have shown the ability to detect abnormal sub-grade conditions, the challenge with using fiber optics rests in obtaining fibers that can resist the installation and severe environments where they can be applied. Non-uniform interactions with the soil and rocks may cause localized strain fields that may not be structurally significant. Methods to separate such behaviors may yield more valuable results in the field. [AASHTO American Association of State and Highway Transportation Officials, 2015 #207].

## Figures and Tables

**Table 1:** Properties of polymers typically used in geogrid materials *.

Polymer	Glass Transition Temperature, Tg (Deg. C)	Density (g/cm^3^)	Modulus of Elasticity (GPa)	Tensile Strength Ultimate, (MPa)
Polyethylene Terephthalate (PET)	70–80	1.38	2.76–4.14	85
High-Density Polyethylene (HDPE)	−125	0.93–0.97	0.65–1.5	26
Low-Density Polyethylene (LDPE)	−125	0.91–0.94	0.19–0.52	10
Polypropylene (PP)	−20 to −5	0.92–0.985	1.14–1.55	9–80
Polyvinyl Chloride (PVC)	87	1.40	0.003–4.14	0.00123–60.8

* The values in this table are for reference only.

**Table 2:** Typical properties from a leading manufacturer of geogrids ^1^ showing the variation of properties across different material classes.

	Property	Uniaxial		Biaxial		Triaxial	
		Low	High	Low	High	Low	High
Index Properties	Rib Pitch (mm)			25	33	33	60
	Mid-Rib Depth or Thickness, (mm)			0.76	1.27	1.2	1.6
	Mid-Rib Width (mm)					0.4	1.2
	Aperture Shape	Higher tensile properties achieved in machine direction only.		Higher tensile properties when loading in the machine or cross-machine directions. Least when loading geogrid at 45 degrees angle to the machine direction		Loads are carried uniformly in all directions. Better in distributing stresses and carrying of axis-loads	
	Tensile Strength @ 5% Strain (kN/m)	14	95	8.5	14.6		
	Ultimate Tensile Strength (kN/m)	35	210	12.4	30		
Structural Durability and Integrity	Junction Efficiency (%)			93	93	93	93
	Flexural Stiffness (mg-cm)	350,000	9,500,000	250,000	750,000		
	Resistance to UV Degradation (%) ^1^	95	95	100	100	70	70

^1^ Polypropylene material.

**Table 3:** Common test standards used to study the properties of the geogrid materials.

Standard Number	Standard Name	Property	Reference
ASTM D4355-21	Standard Test Method for Deterioration of Geotextiles by Exposure to Light, Moisture, and Heat in a Xenon Arc-Type Apparatus	UV oxidation and resistance	[84]
ASTM D6637/D6637M-15	Determining Tensile Properties of Geogrids by the Single or Multi-Rib Tensile Method	Mechanical properties	[17,19,20,31,34,36,43,46,49,65,68]
GRI Test Method GG7 and GG8	Test Method for Carboxyl End Group Content of PET Yarns/Test Method for Determination of the Number Average Molecular Weight of PET Yarns Based on a Relative Viscosity Value	Hydrolysis resistance in PET	[124]
ASTM D5262-07(2016)	Standard Test Method for Evaluating the Unconfined Tension Creep and Creep Rupture Behavior of Geosynthetics,	Creep	[25,34,43,47,48,100]
ASTM D6992-16	Standard Test Method for Accelerated Tensile Creep and Creep-Rupture of Geosynthetic Materials Based on Time-Temperature Superposition Using the Stepped Isothermal Method	Creep	[34,43]
ASTM D4595-17	Standard Test Method for Tensile Properties of Geotextiles by the Wide-Width Strip Method	Mechanical properties	[43,44,48,51,68,81,85]
ASTM D1388-18	Standard Test Method for Stiffness of Fabrics	Mechanical properties	[17]
ASTM D5199-12(2019)	Standard Test Method for Measuring the Nominal Thickness of Geosynthetics	Index properties	[17,48]
ASTM D5261-10(2018)	Standard Test Method for Measuring Mass per Unit Area of Geotextiles	Index properties	[17,34,43,48]
ASTM D7556-10	Standard Test Methods for Determining Small-Strain Tensile Properties of Geogrids and Geotextiles by In-Air Cyclic Tension Tests	Mechanical properties	[44]
ASTM D7737-11	Standard Test Method for Individual Geogrid Junction Strength	Mechanical properties	
ASTM D7748-14	Standard Test Method for Flexural Rigidity of Geogrids, Geotextiles and Related Products	Flexural rigidity	
ISO 10319:2015	Geosynthetics—Wide-Width tensile test	Mechanical properties	[39,44,57,60,63,80,101]
ISO 10722:2007	Geosynthetics—Index test procedure for the evaluation of mechanical damage under repeated loading—Damage caused by granular material	Fatigue	[60]
ISO 11058:2019	Geotextiles and geotextile-related products—Determination of water permeability characteristics normal to the plane, without load	Durability	[60]
ISO 12956:2010	Geotextiles and geotextile-related products—Determination of the characteristic opening size	Index properties	[60]
ISO 13427:2014	Geosynthetics—Abrasion damage simulation (sliding block test)	Durability and installation damage	[60]
ISO 20432:2007	Guidelines for the determination of the long-term strength of geosynthetics for soil reinforcement	Creep	[60]
ISO 10722-1:1998	Geotextiles and geotextile-related products—Procedure for simulating damage during installation—Part 1: Installation in granular materials	Durability and installation damage	[60]
ISO 13431:1999	Geotextiles and geotextile-related products—Determination of tensile creep and creep-rupture behavior.	Creep	
ISO 13438:1999	Geotextiles and geotextile-related products—Screening test method for determining the resistance to oxidation	Thermo-oxidation resistance	
BS EN 20139	Textiles standard atmospheres for conditioning and testing	Durability	[26]
BS 6906	Determination of tensile properties of geosynthetics	Mechanical properties	[27]

## Data Availability

Not applicable.

## Abbreviations

APLT	Automated Plate Load Testing
APT	Accelerated Pavement Testing
BCR	Bearing Capacity Ratio
C&D	Construction and Demolishing
CB	Carbon Black
CBR	California Bearing Ratio
CNT	Carbon Nano Tubes
CRS	Constant Rate of Strain
DCP	Dynamic Cone Penetrometer
DIC	Digital Image Correlation technique
DSC	Digital Scanning Calorimetry
EVAC	Ethylene/vinyl acetate copolymer.
FBG	Fiber Bragg Grating
FTIR	Fourier Transform Infrared
HDPE	High-Density Polyethylene
IRI	International Roughness Index
LDPE	Low-Density Polyethylene
LWD	Lightweight Deflectometer
MD	Machine Direction
PCC	Portland Cement Concrete
PET	Polyethylene Terephthalate
PMMA	Poly (methyl methacrylate)
PP	Polypropylene
PVC	Polyvinyl Chloride
SCR	Stress Concentration Ratio
SEGG	Sensor Enabled Geogrids
SEM	Scanning Electron Microscopy
UV	Ultraviolet
XMD	Cross-Machine Direction
